# Flood Season Microbiota from the Amazon Basin Lakes: Analysis with Metagenome Sequencing

**DOI:** 10.1128/MRA.00229-19

**Published:** 2019-04-25

**Authors:** Célio Dias Santos Júnior, Danyelle Toyama, Tereza Cristina Souza de Oliveira, Fernando Pellon de Miranda, Flávio Henrique-Silva

**Affiliations:** a Laboratório de Biologia Molecular, Departamento de Genética e Evolução, Universidade Federal de São Carlos, São Carlos, São Paulo, Brazil; b Departamento de Química, Universidade Federal do Amazonas, Manaus, Amazonas, Brazil; c Centro de Pesquisas e Desenvolvimento Leopoldo Américo Miguez de Mello, Petróleo Brasileiro S.A. (Petrobras), Rio de Janeiro, Rio de Janeiro, Brazil; Georgia Institute of Technology

## Abstract

Despite an apparent geographic separation of the Amazon water bodies, they are an interconnected system. During floods, the microbiota of rivers, lakes, and soil combines. This study used metagenomics sequencing to survey the microbiota of the Amazon Basin lakes during flood season, showing important patterns in microbial communities.

## Abstract (precis)

Despite an apparent geographic separation of the Amazon water bodies, they are an interconnected system. During floods, the microbiota of rivers, lakes, and soil combines.

## ANNOUNCEMENT

The Amazon Basin is a complex fluvial system reworking almost 3,200 megatons (Mt)/year of floodplain sediment (1), with an annual discharge representing 16% of the freshwater released into the world’s oceans (2). Amazon Basin rivers and lakes constitute two separate systems. However, the flood pulse concept (3, 4) shows a connection between the Amazon River and its floodplain, depending on its water level, which can vary more than 14 m between seasons (5). Previous findings (6, 7) revealed *Actinobacteria*, *Alphaproteobacteria*, *Betaproteobacteria*, *Gammaproteobacteria*, and *Crenarchaeota*, as well as some marine clades (e.g., Ilumatobacter), to be the predominant taxa in the Amazon Basin.

Although there are previous studies on microbial communities in the Amazon lakes, they used samples collected only during the dry season. Therefore, we performed metagenomics high-throughput sequencing to understand the ecology of microbial communities from the lakes throughout the flood season.

Samples from the three lakes were collected in the morning (9:00 a.m. to 12:00 p.m.) in March 2015 (flood season) from Lake Baixio (03°17′4.69″S, 60°03′24.419″W, at a depth of 1 m below the surface), Manacapuru Great Lake (03°19′1.38″S, 60°47′53″W, at a depth of 2 m below the surface), and Lake Preto (03°21′21.76″S, 60°37′36.76″W, at a depth of 1 m below the surface). The sampling procedures and total DNA extraction were conducted as described previously (7). Metadata included the following: for Lake Baixio, temperature of 29.3°C, pH 6.53, turbidity of 24.63 nephelometric turbidity units (NTU), dissolved oxygen (DO) concentration of 2.77 mg/liter, and biological oxygen demand (BOD) of 2.82 mg/liter; for Lake Preto, temperature of 27.8°C, pH 6.2, turbidity of 16.63 NTU, DO concentration of 1.65 mg/liter, and BOD of 1.76 mg/liter; and for Manacapuru Great Lake, temperature of 28.8°C, pH 6.2, turbidity of 19.43 NTU, DO concentration of 5.6 mg/liter, and BOD of 6.1 mg/liter. Environmental DNA libraries were prepared using a Nextera XT DNA kit (Illumina, San Diego, CA, USA) and sequenced with the HiSeq 2500 platform (Illumina). Sequencing yielded 144 million, 132 million, and 180 million paired-end reads of 2 × 100 bp for Lake Baixio, Lake Preto, and Manacapuru Great Lake, respectively. These reads were quality filtered (Q > 20) and assessed for ambiguities using the next-generation sequencing quality control (NGS QC) Toolkit_v2.3.3 (Roche, Indianapolis, IN, USA) (8), utilizing default settings. All high-quality sequences for each metagenome were given functional and taxonomic assignments using the MG-RAST server (Argonne National Laboratory [operated by the UChicago Argonne LLC, Chicago, IL, USA]) (9) at default settings.

The taxonomic composition (percentage of assigned reads) of these metagenomes was 94.0 to 95.2% bacteria, 0.2 to 0.7% archaea, and 0.2 to 0.3% viruses. The phyla observed most often were *Proteobacteria* (abundance, 46.0 to 70.8%), *Actinobacteria* (14.6 to 24.4%), *Cyanobacteria* (2.1 to 13.2%), *Bacteroidetes* (1.2 to 2.0%), and *Firmicutes* (2.1 to 3.4%). Alpha diversity was estimated to be from 281 to 393 species. The functional annotations of the subsystems revealed a predominance of open reading frames (ORFs) related to carbohydrate processing (abundance, 12.7 to 13.5%), protein metabolism (8.6 to 9.0%), and cofactors, vitamins, prosthetic groups, and pigments (7.2 to 7.5%). We observed a predominant ubiquitous genus in all samples, Polynucleobacter (from 1.4 to 9.4%), reinforcing its presence in this ecosystem (6, 7, 10, 11). In summary, this metagenome project reveals initial insight about the Amazon lakes’ microbial communities during the flood season.

### Data availability.

The sequences obtained in this project have been deposited in the European Nucleotide Archive (ENA) under BioProject number PRJEB25171.
